# Dietary Intake and Elevated C-Reactive Protein Levels in US Military Veterans

**DOI:** 10.3390/ijerph18020403

**Published:** 2021-01-06

**Authors:** Stephanie D. Ansley, Jeffrey T. Howard

**Affiliations:** 1 Department of Public Health, University of Texas at San Antonio, San Antonio, TX 78249, USA; jeffrey.howard@utsa.edu; 2 Consequences of Trauma Working Group, the Center for Community-Based and Applied Health Research, University of Texas at San Antonio, San Antonio, TX 78249, USA

**Keywords:** inflammation, nutrition, health behaviors, military health

## Abstract

Elevated inflammatory markers, such as high sensitivity C-reactive protein (hs-CRP), have been associated with the pathogenesis of cardiovascular disease (CVD)-related diseases. However, limited studies have evaluated the potential association between dietary consumption and hs-CRP levels in a large, nationally representative sample, and fewer have investigated their role in ethnic and racial minority military populations. The goal of this study is to explore the relationship between dietary intake, health behaviors, and hs-CRP in individuals with prior military status and whether these associations differ by race/ethnicity. A complex, multistage, probability sample design was used from the National Health and Nutrition Examination (NHANES) 2015–2018 waves. Our results indicate that previously deployed military service members had a higher prevalence of clinically elevated hs-CRP levels than civilians. Differences in hs-CRP among deployed veterans and civilians remained even after multivariable adjustment. Individuals classified as overweight and obese demonstrated clinically elevated hs-CRP levels compared with those with a normal body mass index (BMI). Dietary factors did not attenuate the association between changes in hs-CRP levels and veteran status. These findings suggest the need for further investigation into how military-specific stressors contribute to unfavorable health outcomes for the military population.

## 1. Introduction

In the US, six in ten adults are living with at least one chronic condition [1]. U.S. military service members are not impervious to health conditions found in the general population [2]. For example, cardiovascular disease (CVD) is the leading cause of mortality among military veterans [3]. Even though there has been an increase in US casualty survival rates from combat, cardiac disease (primary/secondary) was the most common diagnosis and primary noninjury cause for critical care transport from deployed settings [4,5].

CVD afflicts military service personnel more than any other chronic disease [6,7,8]. Of concern, the prevalence of hypertension and tobacco use among service members were CVD risk factors that exceeded civilian rates [5,9]. Other modifiable CVD risk factors specific to the military population include physical inactivity, alcohol consumption, illicit drug use, and overweight and obesity status [10,11]. In addition, service members with more exposure to combat trauma during deployments demonstrated higher rates of mental and physical problems and unhealthy behaviors compared to those with less exposure [11]. Systemic inflammatory response syndrome (SIRS), unhealthy behaviors, such as weight gain and decreased physical activity, severity of injuries, and changes in mental well-being have been linked to the development of CVD in US military members [8,12,13,14]. In addition, exposure to stressful and potential life-threating environments exacerbated by mental health issues such as post-traumatic stress disorder (PTSD) converge as prolonged and cumulative stressors for military service members leading to physiological dysregulation through inflammatory pathways [8,15,16,17,18,19,20]. High sensitivity C-reactive protein (hs-CRP) is a biomarker of inflammation and is used to evaluate CVD risk. Clinically elevated hs-CRP levels (i.e., greater than 3.0 mg/L) can be a sign of acute infection, trauma, or chronic disease. CRP levels have been reported to be higher in the military population [21]. Thus, prolonged exposure to stressful environments can lead to “weathering”, a term for accelerated aging, which has been linked to an increased risk of CVD and mortality [22,23,24]. 

In addition to overall increased CVD risks, disparities across race/ethnicity have been reported in the military population as well [13,25,26]. Hispanics and non-Hispanic blacks had a significantly higher prevalence of major CVD risk factors, diabetes and hypertension, compared with non-Hispanic whites [25,26]. Within the Veterans Health Administration (VHA), cardiovascular-related mortality was greater in non-Hispanic blacks than in non-Hispanic Whites, after adjusting for sex and age [27]. The need to understand how stressors contribute to CVD for racial/ethnic groups are underscored by the prediction that by 2040, roughly 34% of all veterans will be from racial/ethnic minority populations [28]. Recent findings by Walker et al. demonstrated that military veterans had greater odds of having CVD than non-veterans [13]. However, the association differed by race/ethnicity such that non-Hispanic black veterans had the same risk as non-veterans while non-Hispanic white veterans had a significantly increased risk of CVD [13].

What is not well understood is the role that inflammation plays in connecting prior military service to subsequent CVD and the extent to which differential inflammatory responses may explain the observed racial/ethnic differences in CVD. The goal of this analysis was to test the hypotheses that (1) prior military service is associated with elevated inflammation, (2) that the military service–inflammation association would differ by race/ethnicity, and (3) dietary factors would attenuate the association between military service and inflammation.

## 2. Materials and Methods

### 2.1. Study Design

The National Health and Nutrition Examination (NHANES) is a research program managed by the National Center for Health and Statistics (NCHS). Since 1999, NHANES has been executed continuously in 2-year waves, which are released to the public as serial, cross-sectional datasets. The NHANES uses a complex, multistage, probability sample design [29]. Among the strengths of the NHANES dataset is that it is a large nationally representative sample that surveys over 5000 individuals every year and that it collects both survey, anthropomorphic, and biological sample data [30]. The survey modules include dietary, demographic, socioeconomic and health-related questions [30]. The examination and biological components consists of medical, dental, and physiological measurements, as well as laboratory tests based on blood and urine samples [30]. Data from the findings are used to determine the prevalence of disease and related risk factors, assess nutritional status, and develop public health policy for Americans [30].

The study protocols of the NHANES were approved by the National Center for Health Statistics Institutional Review Board. Written consent was obtained from all participants. Additionally, the University of Texas at San Antonio Institutional Review Board determined the study to be research not involving human subjects as defined in 45 CFR 46.104(3)(A).

### 2.2. Participants

The interviews and examinations were administered to non-institutionalized US civilians. Mexican Americans, non-Hispanic Blacks, non-Black Asians, low-income whites, and other persons (at or below 185% of the federal poverty level), children 0–11 years, and adults 80 and older were oversampled to increase the reliability and accuracy for these specific subgroups [29,31]. Participants aged 18 years and older who had blood collected for hs-CRP, complete data for the demographic, socioeconomic, health behaviors, dietary factors, and the covariates in the 2015–2018 waves were included in the analysis.

### 2.3. Measures

The outcome measure for this study was the dichotomous variable indicating whether or not each individual’s hs-CRP level exceeded the clinical high-risk threshold of ≥3 (yes or no) [32,33]. The laboratory methodology for the hs-CRP was a two-reagent, immunoturbidimetric system. First, the specimen is combined with a Tris buffer and then incubated. Then, the second reagent (latex particles coated with mouse anti-human CRP antibodies) is added. With the presence of circulating CRP, the latex particles aggregate, which forms immune complexes. These complexes cause an increase in light scattering that is proportional to the CRP concentration. The light absorbance resulting from this light scatter is read against a stored CRP standard curve, which is used to determine the CRP concentration.

Independent variables for this study included demographic, socioeconomic, anthropometric and behavioral measures. Demographic variables included age (as a continuous variable), sex (female or male [reference: the category for each variable to which all other categories are compared]), veteran status (deployed veteran, not deployed veteran, civilian), race/ethnicity (Mexican American, Other Hispanic, non-Hispanic Black, Other Race (including multi-racial), non-Hispanic White [reference]), and marital status (married, widowed, divorced, separated, living with partner, missing, never married [reference]). Socioeconomic variables included income to poverty level (1.0 to 2.0 times poverty, 2.01 to 3.0 times poverty, 3.01 to 4.0 times poverty, more than 4.0 times poverty, missing, and at or below poverty [reference]) and educational attainment (high school graduate or equivalent, some college, college graduate and more, missing, less than high school [reference]). Behavior and anthropometric variables included smoking status (former smoker, current smoker, missing, never smoked [reference]), participation in moderate recreational activities (yes or no), body mass index (BMI) (underweight, overweight, obese, missing, and normal [reference]), and clinical-based high-risk threshold for hs-CRP (low, high).

The computer-assisted dietary interview (CADI) system was used to standardized the interview format to collect the dietary recall data [34]. Energy, nutrients, and non-nutrient foods were estimated from foods and beverages that were consumed during the 24-h period prior to the interview (midnight–midnight) [34]. A multi-pass method interview format was used to collect the dietary information [34]. In-person interviews were conducted in a private setting in the NHANES Mobile Examination Centers (MECs) by trained, bilingual interviewers [34]. A second 24-h dietary recall was conducted via telephone approximately 3–10 days after the MEC exam [35]. The total energy (kcal), carbohydrate (gm), protein (gm), unsaturated fat (gm), and saturated fat (gm) variables were transformed into percentages of total intake for each nutrient. The percent range for the macronutrients were adapted from the Acceptable Macronutrient Distribution Ranges established by the Institute of Medicine (IOM) and the National Academy of Sciences, Engineering and Medicine [36,37]. The IOM recommends adult individuals have 45–65% of their total calories from carbohydrates, 10–30% from protein, and 20–35% from fat [36,37]. To investigate further into the association of dietary fat, unsaturated and saturated fat variables were used. The 2015–2020 Dietary Guidelines for Americans (DGA) suggest consuming less than 10% of total calories from saturated fat [38]. The saturated fat percent ranges were modeled after the DGA guidelines. Recommendations of up to 20–25% of total calories from unsaturated fat provided the adapted ranges for the unsaturated fat category [39].

### 2.4. Statistical Analysis

Descriptive statistics are reported as percentages and standard errors for categorical variables. The unadjusted prevalence of clinically elevated hs-CRP by military veteran status is reported as percentage and 95% confidence intervals (CI). Nested, multivariable logistic regression models were used to analysis the data. The results are reported as Odds Ratios (OR), 95% confidence intervals (CI), and *p*-values. Statistical significance was set at α ≤ 0.05. All analyses were conducted using survey procedures and adjustments to account for population weighting and complex survey design [40]. The IBM^®^ SPSS Statistics PremiumGrad Pack^®^ (version 27.0, IBM, Armonk, NY, USA) was used for all statistical analysis.

## 3. Results

### 3.1. Descriptive Analysis

Demographic and socioeconomic characteristics of 10,736 study participants as weighted percentages are presented in Table 1.

Female and male civilians comprised 56.3% and 43.7% of the population, respectively. Deployed and non-deployed veterans were more men than women (96.8% vs. 3.2%) and (87.8% vs. 12.2%), respectively. More deployed and non-deployed veterans were white (74.9% and 76.0%), had some college education (42.8% and 36.1%), and were married (64.6% and 66.8%). Behavioral and anthropometric variables are presented in Table 2. More deployed and non-deployed veterans were former smokers (49.2% and 45.3%) and obese (49.3% and 42.8%), respectively. Non-deployed veterans participated in more moderate recreational activities (47.8% vs. 44.2%) than deployed veterans. 

**Figure 1:**
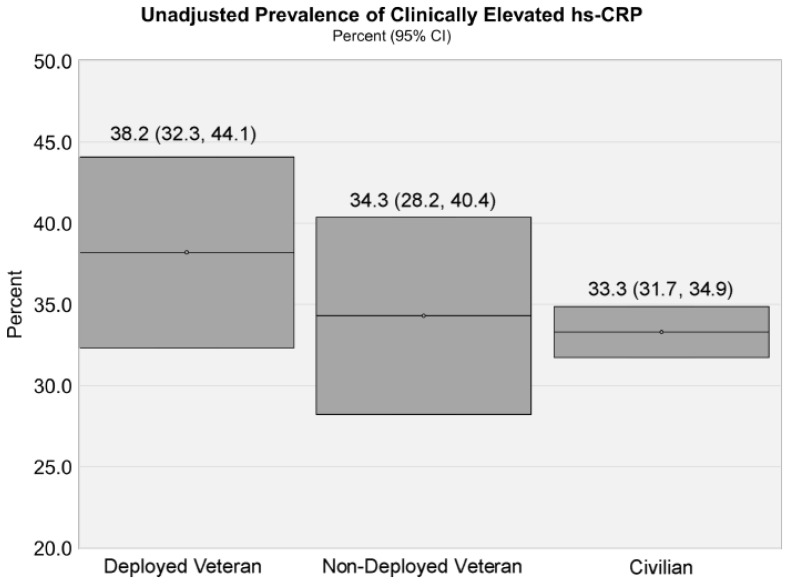
Unadjusted prevalence of clinically elevated high sensitivity C-reactive protein (hs-CRP) levels among veteran status and civilians (10,736). Presented as percentage prevalence and 95% confidence intervals (LCL-UCL). LCL, lower confidence limits; UCL, upper confidence limits.

Mean hs-CRP levels were 4.06 mg/L for deployed veterans, 4.03 mg/L for non-deployed veterans, and 3.87 mg/L for civilians. The unadjusted prevalence of clinically elevated hs-CRP was 38.2% for deployed veterans, 34.3% for non-deployed veterans, and 33.3% for civilians (Figure 1).

### 3.2. Multivariate Analysis

In the age-adjusted logistic regression model, there was not a statistically significant difference in the odds of elevated hs-CRP between veterans and civilians (Table 3, Model 1). After adjusting for demographic, socioeconomic status, nutrition consumption, and health behaviors, associations for deployed veterans and elevated hs-CRP levels remained statistically significant (Table 3, Model 2). Deployed veterans were 1.5 times more likely (OR, 1.48; *p* = 0.007) to have elevated hs-CRP levels than civilians. Females were 1.7 times more likely (OR, 1.68; *p* = 0.000) to have elevated hs-CRP levels than males. Non-Hispanic blacks were 1.2 times more likely (OR, 1.20; *p* = 0.007) to have elevated hs-CRP levels than non-Hispanic whites. Mexican Americans were 1.2 times more likely (OR, 1.23; *p* = 0.015) to have elevated hs-CRP levels than non-Hispanic whites. Individuals with the highest income to poverty ratio were 0.815 as likely (OR, 0.815; *p* = 0.010) to have elevated hs-CRP levels than those at or below poverty level. Married individuals were 1.3 times more likely (OR, 1.32; *p* = 0.001) to have greater CRP levels than those who were not married. Individuals who were separated were 1.4 times more likely (OR, 1.37; *p* = 0.033) to have higher CRP levels than individuals who were not married. Current smokers were 1.3 times more likely (OR, 1.28; *p* = 0.005) to have elevated CRP levels than individuals who had never smoked. Former smokers were 1.3 times more likely (OR, 1.30; *p* = 0.000) to have elevated CRP levels than individuals who had never smoked. Individuals who participated in moderate physical activity were 0.75 as likely (OR, 0.75; *p* = 0.000) to have elevated hs-CRP levels than those who were sedentary.

Individuals who consumed greater than 11% of their total calories from saturated fat were 1.2 times more likely (OR, 1.23; *p* = 0.032) to have elevated hs-CRP levels than those who consumed less than 9% of their total calories from saturated fat (Table 3, Model 2). In addition, individuals who consumed >45–50% of their total calories from carbohydrates were 1.3 times more likely (OR, 1.25; *p* = 0.028) to have elevated hs-CRP levels than those who had consumed 40–45% of their total calories from carbohydrates. Individuals who had less than or equal to 10% of their total calories from protein were 1.8 times more likely (OR, 1.78; *p* = 0.002) to have elevated hs-CRP levels than those who consumed 20–25% of their total calories from protein. No significant associations were observed for educational attainment, unsaturated fat, and calorie intake.

In the fully adjusted model, the association between veteran status and elevated hs-CRP was not meaningfully attenuated by dietary factors (Table 3, Model 3). The odds of elevated hs-CRP levels remained higher in deployed veterans (OR, 1.39; *p* = 0.017). These observations suggest that military service, specifically deployment, and dietary factors act as independent risk factors. Females showed significantly higher odds (OR, 1.78; *p* = 0.000) of having elevated hs-CRP than males. Individuals at the more than 4.0 times poverty level were 0.77 as likely (OR, 0.768, *p* = 0.001) to have elevated hs-CRP values than those at or below poverty level.

Married individuals were 1.3 times more likely (OR, 1.27; *p* = 0.006) to have greater CRP levels than those who were not married. Current smokers were 1.4 times more likely (OR, 1.42; *p* = 0.000) to have elevated CRP levels than individuals who had never smoked. Former smokers were 1.3 times more likely (OR, 1.25; *p* = 0.003) to have elevated CRP levels than individuals who had never smoked. Individuals who participated in moderate physical activity were 0.84 as likely (OR, 0.84; *p* = 0.029) to have elevated hs-CRP levels than those who were sedentary. No significant associations were observed for race/ethnicity and educational attainment.

Individuals who consumed >45–50% of the total calories from carbohydrates were 1.2 times more likely (OR, 1.24; *p* = 0.044) to have elevated hs-CRP levels than individuals who consumed 40% thru 45% of total calories from carbohydrates. Individuals who had less than or equal to 10% of their total calories from protein were roughly two times more likely (OR, 1.97; *p* = 0.000) to have elevated hs-CRP values than individuals who consumed 20–25% of their total calories from protein. Overweight individuals were two times more likely (OR, 2.02; *p* = 0.000) to have elevated hs-CRP levels than individuals with a normal BMI. Obese individuals had a 6-fold increase in odds (OR, 5.94; *p* = 0.000) of having elevated hs-CRP levels compared to those with a normal BMI. No significant associations were observed for calorie intake, saturated, and unsaturated fat consumption.

**Figure 2:**
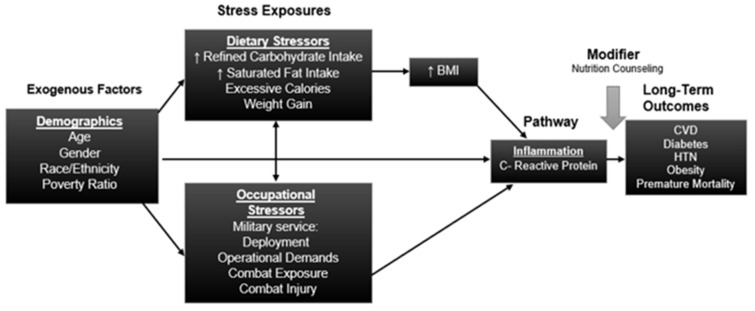
Conceptual model of stress exposures, pathway, and long-term outcomes. BMI indicates body mass index, CVD indicates cardiovascular disease, HTN indicates hypertension.

In addition, the fully adjusted model highlights how the OR for other dietary factors were weakened, after adjusting for BMI. For example, in Table 3, Model 2, individuals who consumed greater than 11% of their total calories from saturated fat were 1.2 times more likely to have elevated hs-CRP levels than individuals who consumed less than 5–9% of their total calories from saturated fat. However, this variable became a nonsignificant predictor of elevated hs-CRP levels when BMI was accounted for. In summary, dietary factors have an impact on the odds of having elevated hs-CRP values, but the influence operates through BMI, as seen in Figure 2. Tests of interaction between veteran status and race/ethnicity were also not statistically significant.

## 4. Discussion

Our findings support the primary hypothesis that military veterans who were deployed have a higher prevalence of clinically elevated hs-CRP levels than civilians. These results persisted even after adjusting for demographic, socioeconomic, and health behavior risk factors. Additionally, we tested the interaction between military service and race/ethnicity and found that the military service–hs-CRP association did not differ significantly by race/ethnicity. Previous research reported a significant association with longer deployment periods and greater hs-CRP levels in military members [21]. Stressful environments encountered during military operations such as lengthy and repeated deployments, operational demands, and exposure to combat are chronic stressors that can be responsible for the increased amounts of circulating pro-inflammatory cytokines and the inflammatory processes [8,21,41,42]. Our results are consistent with previous research that reported significantly elevated hs-CRP levels in veterans with a diagnosis of Gulf War Illness (GWI), which further supports a potential mechanism that chronic inflammation is an influential component to the pathophysiology of multi-system disorders related to specific military situations such as combat exposure [41]. Systemic chronic inflammation has been implicated in the development and progression of chronic diseases [42]. For example, inflammation and endothelial dysfunction due to the stress-induced dysregulation of the sympathetic nervous system and the hypothalamic–pituitary–adrenal axis can increase the risk of hypertension and other cardiovascular diseases [8,17,43,44,45].

Although we did not study the differences between genders, our results show that females were more likely to have clinically elevated hs-CRP levels than males. Biological differences could be a possible explanation in the association between gender and CRP levels [46,47,48]. For example, researchers demonstrated that exogenous hormone therapy elevated CRP levels in women [47,48]. Therefore, it is plausible greater CRP levels could be attributed to higher estrogen levels in females [46]. Ishii et al. highlighted gender differences in the obesity–inflammation association and elevated CRP levels [46]. Women tend to have a greater body fat percentage than men [49,50]. Thus, a greater accumulation of subcutaneous fat in women can partially explain their higher CRP values [46,49].

We observed that individuals with higher incomes were less likely to have elevated hs-CRP levels compared to individuals at or below the poverty level. Previous research support this finding. After adjusting for potential confounding variables, socioeconomic status (SES) demonstrated an inverse relationship with hs-CRP levels [51]. Of concern, every decrease in a socioeconomic position is associated with an increase in the prevalence of disease, such as cardiovascular disease [52,53]. The military population is not immune to health disparities due to SES. Military rank is a reliable indicator of SES and income status [54]. Researchers found military officers had lower odds of reporting poor or fair health compared to enlisted members [55].

Our study demonstrated a statistically significant association between moderate physical activity and lower odds of having elevated hs-CRP levels. One potential mechanism is that moderate physical activity has been shown to decrease the inflammatory cytokine production resulting in chronic body inflammation [56,57].

No statistically significant association in education was observed in our study. Elevated hs-CRP remained statistically associated with current and former smokers after being fully adjusted for additional health behaviors. These findings are consistent with previous research linking cigarette smoking to increases in CRP levels [58,59]. Exposure to cigarette smoke increases oxidative stress and consequently vascular inflammation [60]. Married individuals were more likely to have clinically elevated hs-CRP levels than individuals who were not married. These findings were contrary to previous research demonstrating marriage was a protective factor against elevated CRP values [61]. Further research into spousal support and overall wellness status should be investigated to further explore the association between marital status and CRP levels.

Race/ethnicity could not explain the differences in hs-CRP levels among veterans and civilians. Although limited research has been reported on differences in hs-CRP values in minority veterans, previous studies have shown racial/ethnic disparities in health outcomes among the military population. As for US Air Force active duty service members, non-Hispanic blacks were significantly more likely to be diagnosed with hypertension, dyslipidemia, and diabetes compared to non-Hispanic whites after adjustment for sex and rank [25]. Furthermore, Hispanics and non-Hispanic blacks had significantly greater prevalence of diabetes compared to non-Hispanic whites in all age categories [25]. Black, Hispanic, and other race veterans were significantly more likely to report poorer self-rated health compared to White veterans after controlling for age, socioeconomic status, smoking, and military experiences [62]. These studies are in contrast to findings that non-Hispanic whites with prior military service had a greater age-adjusted prevalence of CVD compared to non-Hispanic blacks and Hispanics, regardless of previous military service [13]. The researchers noted that military service can provide upward mobility in income and SES, resulting in protective buffers to CVD for racial/ethnic minorities [13]. Our results were not consistent with a differential inflammatory process explanation for the observations by Walker et al. [62]. However, they are consistent with the overall hypothesis that military service and deployment is associated with increased inflammation, which may be induced by increased exposure to stressful environments.

Dietary factors did not substantively attenuate the association between changes in hs-CRP levels and veteran status. Instead, these data suggest that dietary factors may act as independent risk factors mediated through BMI status. This indicates that the dietary factors played a role in determining BMI, which ultimately is independently associated with inflammation.

A healthier diet has the potential to interrupt this inflammatory pathway and improve the risk of CVD among the military population. In a prior study, obese individuals had statistically significant improvements in their hs-CRP profile with adherence to a low-calorie, Mediterranean-style diet [63]. Fruits, vegetables, and whole grain consumption were inversely associated with hs-CRP in a multi-ethnic adult study population [64]. On the contrary, the Western diet, characterized by excessive calories, high in saturated fat and refined carbohydrates, promotes a pro-inflammatory response and subsequently elevated hs-CRP levels [65,66,67]. In the military population, extended hours, rotating shifts, irregular mealtimes, and limited healthy food selections during mission training and deployment can expose military personnel to additional noncombat environmental stressors such as diet [68,69].

In our study, individuals with obese and overweight status were more likely to have elevated hs-CRP levels than those with a normal BMI. These findings are consistent with previous research demonstrating elevated hs-CRP levels in overweight and obese US adults from a nationally representative sample [70]. An overweight and obese prevalence has been studied in the military sector. Service members reportedly gain an average 0.6–0.7 kg/yr. during their service, and the rate hastens around the time of their discharge [71,72]. Furthermore, roughly 44% of younger veterans are obese [73]. We also found that a low consumption of protein (≤10% of total calories) and higher consumption of carbohydrates (>45–50% of total calories) was associated with increased odds of elevated hs-CRP compared to individuals who consumed the recommended amount of protein (20–25% of total calories) and carbohydrates (40–45% of total calories), even when BMI was included in the final model. This may simply reflect the fact that if less protein is being consumed, then it is likely that greater amounts of refined carbohydrates are being consumed. These results suggest that closer adherence to dietary guidelines for total protein, carbohydrate, and fat consumption may result in lower system inflammation, all other things being equal.

Since hs-CRP is a reliable predictor of CVD, it is important to further explore military-specific stressors contributing to unfavorable health outcomes for our military population. Future research should consider hs-CRP differences in the female active duty and veteran groups, especially in more recent cohorts of female service members who may have more exposure to combat environments. In 2015, policy changes allowed the military to open all combat positions and units to women without exceptions; however, it is likely that many military veterans in our sample served prior to this time [74]. Additionally, poor diet may operate as a daily environmental stressor and a major contributor to chronic conditions such as CVD. Future research should investigate the effectiveness of an anti-inflammatory diet, such as the Mediterranean-style eating pattern, on cardiometabolic outcomes and changes in inflammatory markers among military members. Approximately 60% of Armed Forces personnel report regular consumption of dietary supplements [75,76]. Adverse events associated with dietary supplements can be exacerbated by military-specific stressors [75,76]. Therefore, more education and monitoring related to dietary supplement use in the military community are warranted [75,76].

## 5. Strengths and Limitations

Our study is not without limitations. NHANES is a cross-sectional study, which limits the ability to attribute a causal relationship. The limited NHANES data did not allow us to examine additional military-related factors such as the length of deployment and service, repeated deployments, the branch of service, military rank, war era, and combat-related exposures such as injuries. A cross-sectional study does not reflect past nutritional consumption. Individuals’ dietary intake can vary daily. Limited dietary data did not allow for a complete assessment of adherence to a specific diet, such as the Mediterranean, or consumption of anti- and pro-inflammatory foods/beverages. Longitudinal studies that examined dietary patterns would further explain the role diet has on hs-CRP levels. Inaccurate reporting or poor memory of participants can raise concerns about the accuracy of the dietary data. Although the interviewers were trained on the multiple pass method, measurement error can still occur. It is worth mentioning that because BMI is an indirect measure of body fat; muscular military personnel might have been misclassified as overweight or obese. Studies using alternative anthropometric methods (e.g., bioelectrical impedance and skinfold thickness) could further explain the association between body composition and hs-CRP levels.

## 6. Conclusions

Our results support an association between status as a deployed military veteran and clinically elevated hs-CRP levels.

## Figures and Tables

**Table 1:** Weighted demographic and socioeconomic characteristics (*n* = 10,736).

Variables	Deployed Veteran (*n* = 524)	Non-Deployed Veteran (*n* = 505)	Civilian (*n* = 9707)
Age, percent (SE)			
18–24	0.1 (0.1)	3.5 (1.3)	9.6 (0.5)
25–34	8.5 (1.7)	6.6 (1.6)	19.8 (0.7)
35–44	12.2 (2.1)	7.7 (1.8)	17.2 (0.8)
45–54	15.3 (2.4)	13.4 (2.2)	17.6 (0.7)
55–64	11.2 (2.5)	21.8 (3.0)	18.3 (0.7)
65 and older	52.8 (3.4)	47.0 (3.7)	17.5 (0.8)
Sex, percent (SE)			
Male	96.8 (1.0)	87.8 (2.2)	43.7 (0.6)
Female	3.2 (1.0)	12.2 (2.2)	56.3 (0.6)
Race/Ethnicity, percent (SE)			
Non-Hispanic White	74.9 (2.8)	76.0 (2.6)	61.8 (2.4)
Non-Hispanic Black	11.0 (2.0)	11.4 (1.6)	11.4 (1.4)
Mexican American	2.5 (0.4)	3.7 (1.1)	9.4 (1.4)
Other Hispanic	4.2 (1.1)	2.5 (0.6)	7.0 (0.8)
Other (including multi-racial)	7.4 (1.6)	6.4 (1.0)	10.3 (1.0)
Education, percent (SE)			
Less than High School	5.8 (1.2)	7.0 (1.1)	13.6 (1.0)
High School Graduate or Equivalent	21.2 (2.5)	28.1 (2.8)	23.9 (1.0)
Some College	42.8 (2.7)	36.1 (2.9)	30.8 (1.0)
College Degree or More	30.2 (3.1)	28.8 (2.7)	31.7 (2.0)
Missing			0.1 (0.0)
Marital Status, percent (SE)			
Never Married	5.7 (1.4)	8.1 (2.4)	19.6 (0.8)
Married	64.6 (2.7)	66.8 (3.9)	52.5 (1.2)
Live with Partner	5.2 (1.4)	5.2 (1.1)	9.9 (0.6)
Widowed	8.2 (1.5)	5.1 (0.9)	5.8 (0.3)
Separated	3.3 (1.2)	2.9 (1.2)	2.6 (0.2)
Divorced	13.1 (2.4)	11.8 (2.1)	9.7 (0.5)
Refused			0.0 (0.0)
Income to Poverty Ratio, percent (SE)			
At or Below Poverty	14.8 (1.9)	13.9 (2.7)	22.7 (1.1)
1.0 to 2.0 Times Poverty	17.6 (1.7)	19.8 (2.5)	18.7 (0.9)
2.01 to 3.0 Times Poverty	14.6 (2.4)	16.5 (2.4)	14.3 (0.9)
3.01 to 4.0 Times Poverty	15.5 (2.3)	11.1 (2.2)	10.9 (0.7)
More than 4.0 Times Poverty	37.5 (2.5)	38.6 (3.5)	33.4 (1.7)

**Table 2:** Weighted behavioral, anthropometric, and clinical characteristics (*n* = 10,736).

Variables	Deployed Veteran (*n* = 524)	Non-Deployed Veteran (*n* = 505)	Civilian (*n* = 9707)
Smoking Status, percent (SE)			
Current Smoker	17.2 (2.1)	16.8 (2.1)	18.0 (0.8)
Former Smoker	49.2 (3.0)	45.3 (2.6)	22.7 (0.7)
Never Smoker	33.6 (3.0)	37.9 (2.5)	59.2 (1.0)
Missing		0.1 (0.1)	0.1 (0.0)
Moderate Physical Activity, percent (SE)			
Yes	44.2 (3.5)	47.8 (3.3)	46.7 (1.4)
No	55.8 (3.5)	52.1 (3.3)	53.2 (1.4)
Missing			0.0 (0.0)
Body Mass Index, percent (SE)			
Underweight	0.5 (0.3)	1.2 (0.6)	1.5 (0.2)
Normal weight	15.7 (2.0)	17.2 (2.7)	26.4 (1.0)
Overweight	33.4 (2.5)	37.0 (4.5)	30.7 (0.7)
Obese	49.3 (3.2)	42.8 (3.1)	40.2 (1.2)
Missing	1.1 (0.4)	1.9 (0.7)	1.2 (0.1)
Nutrient Consumption, percent (SE)			
Carbohydrate			
Missing/Unknown	15.5% (2.3%)	14.5% (1.9%)	19.1% (0.8%)
Less than 40%	22.9% (2.7%)	21.4% (2.1%)	19.0% (0.8%)
40% through 45%	18.9% (2.2%)	21.0% (2.8%)	15.7% (0.7%)
>45% through 50%	16.8% (2.5%)	16.5% (2.6%)	17.4% (0.6%)
>50% through 55%	12.4% (2.4%)	14.0% (1.0%)	15.1% (0.6%)
Greater than 55%	13.4% (2.5%)	12.5% (2.1%)	13.7% (0.5%)
Protein			
Missing/Unknown	15.5% (2.3%)	14.5% (1.9%)	19.1% (0.8%)
< or equal to 10%	3.6% (1.0%)	3.5% (0.9%)	3.9% (0.2%)
>10% through <15%	39.4% (3.7%)	36.3% (3.9%)	32.9% (1.0%)
15% through 20%	32.8% (3.4%)	35.0% (2.9%)	31.8% (0.7%)
20% through 25%	5.8% (1.0%)	7.8% (2.2%)	9.4% (0.4%)
Greater than 25%	2.8% (1.0%)	2.8% (1.3%)	2.9% (0.3%)
Saturated Fat			
Missing/Unknown	15.5% (2.3%)	14.5% (1.9%)	19.1% (0.8%)
Less than 5%	0.1% (0.0%)	0.4% (0.3%)	1.5% (0.2%)
5% through 9%	12.5% (1.9%)	11.3% (1.4%)	14.9% (0.5%)
>9% through 11%	18.1% (2.2%)	14.1% (2.5%)	18.4% (0.7%)
Greater than 11%	53.8% (3.1%)	59.7% (2.5%)	46.1% (0.9%)
Unsaturated Fat			
Missing/Unknown/≤0	15.5% (2.3%)	14.5% (1.9%)	19.1% (0.8%)
>0% through 15%	1.6% (0.6%)	2.4% (0.7%)	3.5% (0.2%)
>15% through 20%	12.2% (2.1%)	9.2% (1.7%)	13.9% (0.7%)
>20% through 25%	28.8% (3.2%)	29.8% (2.3%)	29.8% (0.7%)
Greater than 25%	41.8% (3.0%)	44.0% (2.8%)	33.8% (0.9%)
Calories			
Missing/Unknown	15.5% (2.3%)	14.5% (1.9%)	19.1% (0.8%)
<800	1.1% (0.5%)	0.7% (0.3%)	2.0% (0.2%)
≥800 and <1200	4.1% (0.8%)	4.4% (1.2%)	7.6% (0.5%)
≥1200 and <1500	8.4% (1.7%)	6.0% (1.3%)	11.5% (0.5%)
≥1500 and <1800	11.5% (2.1%)	11.3% (2.0%)	13.3% (0.4%)
≥1800 and <2000	12.6% (2.0%)	11.6% (2.4%)	8.6% (0.4%)
≥2000 and <2200	10.4% (1.7%)	9.5% (1.8%)	8.1% (0.5%)
≥2200 and <2500	11.1% (1.9%)	12.4% (2.6%)	9.4% (0.3%)
2500 and greater	25.3% (3.4%)	29.6% (2.8%)	20.4% (0.7%)
Clinical based high-risk threshold for hs-CRP			
Low	61.8% (3.0%)	65.7% (3.1%)	66.7% (0.8%)
High	38.2% (3.0%)	34.3% (3.1%)	33.3% (0.8%)

**Table 3:** Weighted results adjusted for age, demographic, socioeconomic status (SES), and health behaviors (*n* = 11,285).

Variable	Model 1 Age-Adjusted Only OR (95% CI); *p*-Value	Model 2 Adjusted for Demographic, SES, Nutrition and Health Behaviors OR (95% CI); *p*-Value	Model 3 Fully Adjusted w/BMI OR (95% CI); *p*-Value
Military Service			
Deployed Veteran	1.16 (0.89, 1.52); 0.249	1.48 (1.13, 1.96); 0.007	1.39 (1.06, 1.82); 0.017
Non-deployed Veteran	0.98 (0.75, 1.30); 0.931	1.20 (0.89, 1.64); 0.226	1.20 (0.85, 1.69); 0.285
Civilian (ref)			
Age		1.00 (1.00, 1.01); 0.670	1.00 (1.00, 1.00); 0.894
Sex			
Women		1.68 (1.43, 1.99); 0.000	1.78 (1.51, 2.12); 0.000
Men (ref)			
Race/Ethnicity			
Non-Hispanic White (ref)			
Non-Hispanic Black		1.20 (1.06, 1.37); 0.007	1.05 (0.91, 1.22); 0.450
Mexican American		1.23 (1.05, 1.47); 0.015	1.01 (0.85,1.20); 0.896
Other Hispanic		1.23 (0.98,1.55); 0.073	1.15 (0.91,1.47); 0.222
Other		0.871 (0.71, 1.06); 0.169	0.989 (0.79, 1.23); 0.919
Education			
Less than High School (ref)			
High School Graduate or			
Equivalent		1.14 (0.95,1.37); 0.141	1.06 (0.88,1.29); 0.517
Some College		1.08 (0.93, 1.26); 0.289	0.977 (0.83, 1.15); 0.769
College Degree or More		0.872 (0.71, 1.07); 0.173	0.935 (0.75,1.17); 0.542
Missing/Unknown		0.473 (0.18, 1.24); 0.123	0.384 (0.10, 1.46); 0.155
Marital Status			
Never Married (ref)			
Married		1.32 (1.13, 1.56); 0.001	1.27 (1.08, 1.50); 0.006
Live with Partner		1.21 (0.96, 1.53); 0.100	1.15 (0.93, 1.44); 0.191
Widowed		1.00 (0.74, 1.37); 0.966	0.941 (0.70, 1.27); 0.681
Separated		1.37 (1.03, 1.84); 0.033	1.27 (0.95, 1.70); 0.099
Divorced		1.13 (0.91, 1.42); 0.260	1.04 (0.82, 1.31); 0.734
Refused		2.53 (0.30, 21.25); 0.378	3.09 (0.17, 56.57); 0.433
Income to Poverty Ratio			
At or Below Poverty (ref)			
1.0 to 2.0 Times Poverty		1.00 (0.86, 1.16); 1.00	1.00 (0.85, 1.19); 0.925
2.01 to 3.0 Times Poverty		0.973 (0.84, 1.12); 0.703	0.941 (0.82, 1.09); 0.397
3.01 to 4.0 Times Poverty		1.04 (0.87, 1.27); 0.618	1.01 (0.82, 1.25); 0.884
More than 4.0 Times Poverty		0.815 (0.70, 0.95); 0.010	0.768 (0.66, 0.89); 0.001
Missing			
Smoking Status			
Never Smoker (ref)			
Current Smoker		1.28 (1.08, 1.52); 0.005	1.42 (1.21, 1.69); 0.000
Former Smoker		1.30 (1.17, 1.47); 0.000	1.25 (1.09, 1.45); 0.003
Missing		1.21 (0.22, 6.64); 0.818	1.11 (0.11, 11.04); 0.921
Body Mass Index			
Normal weight (ref)			
Underweight			0.576 (0.23,1.43); 0.223
Overweight			2.02 (1.73, 2.36); 0.000
Obese			5.94 (4.92, 7.19); 0.000
Missing			5.89 (3.03, 11.45); 0.000
Moderate Recreational Activity			
Yes		0.758 (0.66, 0.88); 0.000	0.847 (0.73, 0.98); 0.029
No (ref)			
Missing/Unknown		2.43 (0.29, 20.64); 0.403	4.09 (0.53, 31.80); 0.170
Saturated Fat Consumption			
Less than 5%		0.715 (0.43, 1.19); 0.189	0.908 (0.55, 1.50); 0.694
5% through 9% (ref)			
>9% through 11%		0.987 (0.80, 1.22); 0.899	1.00 (0.79, 1.28); 0.966
Greater than 11%		1.23 (1.02, 1.48); 0.032	1.16 (0.95,1.42); 0.137
Unsaturated Fat Consumption			
>0% through 15%		1.25 (0.86, 1.82); 0.227	1.28 (0.90, 1.84); 0.164
>15% through 20% (ref)			
>20% through 25%		0.994 (0.82, 1.20); 0.952	0.968 (0.81, 1.17); 0.725
Greater than 25%		1.15 (0.93, 1.44); 0.185	1.06 (0.87, 1.30); 0.548
Carbohydrate Consumption			
Less than 40%		1.10 (0.91, 1.36); 0.307	1.06 (0.85, 1.33); 0.584
40% through 45% (ref)			
>45% through 50%		1.25 (1.03, 1.54); 0.028	1.24 (1.00, 1.54); 0.044
>50% through 55%		1.27 (1.00, 1.63); 0.054	1.25 (0.97, 1.62); 0.085
Greater than 55%		1.14 (0.88, 1.50); 0.315	1.12 (0.84, 1.52); 0.426
Protein Consumption			
<or equal to 10%		1.78 (1.25, 2.54); 0.002	1.97 (1.39, 2.81); 0.000
>10% through <15%		1.19 (0.89, 1.61); 0.239	1.34 (0.98, 1.85); 0.066
15% through <20%		1.09 (0.86, 1.40); 0.450	1.17 (0.91, 1.50); 0.202
20% through 25% (ref)			
Greater than 25%		0.888 (0.61, 1.28); 0.514	0.849 (0.57, 1.28); 0.417
Calories Consumption			
Missing/Unknown		1.28 (0.80, 2.05); 0.289	1.40 (0.84, 2.36); 0.186
<800		1.16 (0.88, 1.54); 0.268	1.22 (0.83, 1.82); 0.296
≥800 and <1200		1.13 (0.84,1.51); 0.399	1.15(0.81, 1.63); 0.418
≥1200 and <1500		0.989 (0.77, 1.27); 0.927	1.06 (0.82, 1.39); 0.608
≥1500 and <1800		0.815 (0.61, 1.09); 0.158	0.873 (0.64, 1.19); 0.383
≥1800 and <2000 (ref)			
≥2000 and <2200		0.906 (0.69, 1.20); 0.478	0.940 (0.72, 1.23); 0.645
≥2200 and <2500		0.808 (0.57, 1.15); 0.229	0.853 (0.59, 1.23); 0.379
2500 and greater		0.869 (0.67, 1.13); 0.290	0.872 (0.66, 1.16); 0.332

## Data Availability

All data used in this study are publicly available from the US Centers for Disease Control and Prevention (CDC), at the following websites: (1) for 2015–2016 data https://wwwn.cdc.gov/nchs/nhanes/continuousnhanes/default.aspx?BeginYear=2015, (2) for 2017–2018 data: https://wwwn.cdc.gov/nchs/nhanes/continuousnhanes/default.aspx?BeginYear=2017.
